# Bilateral Sylvian fissure lipomas with angiomatous component: A case report

**DOI:** 10.1016/j.radcr.2025.05.110

**Published:** 2025-06-26

**Authors:** Mireille M. de Vries, Nadine Oosterhof, Carine Martins Jarnalo, Marjolein Dremmen, Janet de Beukelaar, Nienke Katier

**Affiliations:** a Department of Neurology, Albert Schweitzer Hospital, Dordrecht, The Netherlands; b Department of Radiology, Albert Schweitzer Hospital, Dordrecht, The Netherlands; c Department of Radiology and Nuclear Medicine, Erasmus MC University Medical Center, Rotterdam, The Netherlands

**Keywords:** Sylvian fissure, Intracranial lipoma, Bilateral, Angiomatous component, Coincidental finding

## Abstract

Sylvian fissure lipomas are rare congenital malformations, with few cases reported in the literature. We present the case of a 28-year-old woman who consulted a neurologist for headache during pregnancy. Brain magnetic resonance imaging (MRI) showed bilateral extra-axial perisylvian lesions as an incidental finding. The MRI characteristics led to the diagnosis of bilateral Sylvian fissure lipomas with angiomatous component. To our knowledge, no previous cases of bilateral Sylvian fissure lipomas have been reported. This article provides an overview of the literature on Sylvian fissure lipomas, including their prevalence, pathophysiology, differential diagnosis, and management, to aid in their timely recognition and appropriate evaluation.

## Introduction

Incidental findings are common on brain magnetic resonance imaging (MRI) and may result in substantial resource expenditure and patient anxiety. Literature on incidentalomas assists radiologists in determining the nature of these lesions within a short time frame and provides the clinician with background information for patient management.

We present a 28-year-old patient with the coincidental finding of bilateral fat-containing, intracranial, extra-axial lesions in the Sylvian fissure. The location and bilaterality suggest a congenital etiology. The differential diagnosis includes intracranial lipomas, dermoid cysts, and lipomatous meningiomas. All 3 entities are rare, and have some overlapping features on MRI. Based on MRI characteristics, we diagnosed the lesions as bilateral Sylvian fissure lipomas with angiomatous component.

To our knowledge, this is the first reported case of bilateral Sylvian fissure lipomas with angiomatous component. We provide an overview of the literature concerning Sylvian fissure lipomas, including prevalence, pathophysiology, differential diagnosis, management, and associated pathology.

### Case

**Fig. 1:**
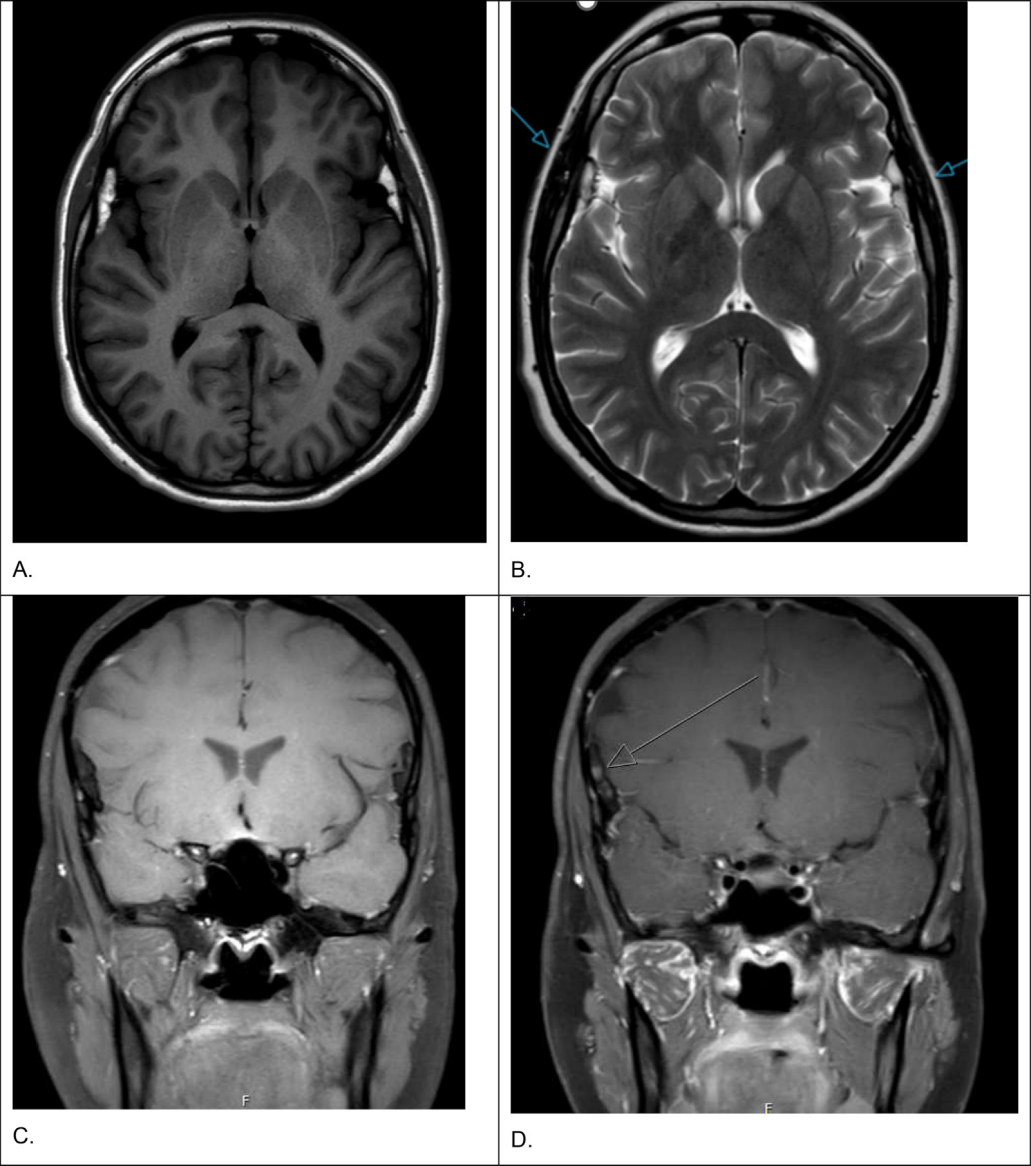
MRI of the bilateral sylvian fissure lipomas with angiomatous component. MR images at the level of the Sylvian fissure with axial T1-weighted sequence (A), axial T2-weighted sequence (B), coronal pre- and postcontrast T1-weighted sequence with fat suppression technique (C and D). The images show bilateral perisylvian lesions with hyperintense signal on the T1-weighted sequence (A), with hypointense signal with fat suppression technique on the T1-weighted sequence (C and D) indicating that the lesions contain fat. On the T2-weighted sequence the lesions are also hyperintense (B). After administration of gadolinium, the lesions show partially enhanced signal (arrow) on T1-weighted imaging, indicating an angiomatous component of the lesions (D).

A 28-year-old woman presented to our Neurology outpatient clinic with a severe bilateral frontal headache during the third trimester of her pregnancy. She had been experiencing a pulsating, and dull, persistent headache for 3 days, with increasing severity. She did not experience any visual disturbances, but she occasionally experienced a tingling sensation in her hands. Her medical history included migraine (with her last episode occurring several years ago) and previous gastric bypass surgery. Her family history included a grandmother diagnosed with migraine with aura. On examination she was afebrile, normotensive, and without peripheral edema or proteinuria; neurological assessment was normal with no signs of papilledema, and laboratory tests revealed hemoglobin 6.5 mmol/L with normal renal and liver function. Obstetric and gynecological evaluations were unremarkable. Brain MRI was performed to exclude serious causes of headache like posterior reversible encephalopathy syndrome (PRES) and cerebral venous sinus thrombosis (CVST). MR brain images did not show PRES, CVST, or other acute causes of her headache. However, it revealed as a coincidental finding 2 symmetric intracranial, extra-axial lesions at the Sylvian fissure. The location and bilaterality suggested a congenital etiology, and follow-up was recommended (Fig. 1).

To assess the lesions for growth over time, a follow-up brain MRI was scheduled 3 months later, but was postponed several times due to personal circumstances of the patient. Finally, 17 months later, a follow-up MRI and magnetic resonance angiography (MRA) were performed with additional sequences. The volume of the lesions remained unchanged. MRI showed lesions with hyperintense signal intensity and a hypointense rim on T1-and T2-weighted sequences. On fat-suppressed T2 Short TI Inversion Recovery (STIR) sequence, the lesions turned hypointense. Diffusion weighted imaging (DWI) did not show diffusion restriction. After gadolinium administration, both lesions showed linear/tubular enhancement, correlating to an intralesional vascular structure on MRA. Susceptibility-weighted imaging (SWI) demonstrated extensive blooming artifacts. This combination of MRI characteristics confirmed the lesions as fatty with an angiomatous component.

Our patient was diagnosed with migraine and tension-type headache, and treated conservatively. Her headache spontaneously decreased in severity during follow up. We concluded that the most probable radiological diagnosis of the intracranial lesions is bilateral Sylvian fissure lipomas with angiomatous component. The patient was informed about this diagnosis and its rarity, and she provided written informed consent to publish her data anonymously.

### The differential diagnosis of fat-containing, intracranial, extra-axial lesions

The differential diagnosis of congenital, fat-containing, intracranial, extra-axial lesions includes lipomatous meningiomas, dermoid cysts, and intracranial lipomas. Teratomas were not considered due to the absence of other components of the 3 germ cell layers [[1], [2], [3]]. Immature teratomas can contain only fat but are typically seen in young children [2]. All 3 entities are rare and have overlapping features on MRI (Table 1).

**Table 1:** MRI characteristics of fatty intracranial lesions in adults: Intracranial lipomas (with or without angiomatous component), dermoid cysts and lipomatous meningiomas.

	Lipomas (with or without angiomatous component)	Dermoid cysts	Lipomatous meningiomas
MRI			
T1	Hyperintense	Hyperintense	Hyperintense
T2	Hyperintense	Heterogenous	Hyperintense
Fat-suppression	Hypointense	Hypointense	Hypointense
DWI/ADC	No diffusion restriction	No diffusion restriction	No diffusion restriction
Postcontrast	No enhancement (or partial enhancement in angiomatous variant)	No enhancement	Homogenous to heterogenous enhancement
Characteristic feature			Dural tail

Lipomatous meningiomas are solid lesions with a broad dural attachment (dural tail), containing adipocyte-like cells. When lipomatous meningiomas contain a high amount of fat, they are T1 and T2 hyperintense, with suppressed signal on fat-suppression sequences. Unlike the lesions in this case, lipomatous meningiomas typically show homogenic enhancement, but heterogeneous enhancement has been described. No diffusion restriction has been reported [4,5].

Intracranial dermoid cysts contain desquamated squamous epithelium, hair follicles, sweat glands and sebaceous glands [6]. These lesions are hyperintense on T1-weighted images, and heterogeneous on T2-weighted images. They can cause blooming artifacts on susceptibility weighted sequences due to calcifications [5]. They show no diffusion restriction and characteristically no enhancement [5,7]. Intracranial dermoid cysts are typically located in the midline or in regions of sutures (often intracranial expansion of extracranial dermoid) [6].

Intracranial lipomas show T1 and T2 hyperintensity with suppressed signal on fat-suppressed sequences. Possible curvilinear or nodular calcifications may appear as T1-hyperintensities [5]. The angiomatous variant, with vessels or nerves through the lesion, is extremely rare [5,[8], [9], [10], [11]]. Intracranial lipomas create blooming artifacts on susceptibility weighted images in case of calcifications [12].

Teratomas are rare central nervous germ cell tumors (GCT) that develop during embryogenesis and comprise elements from all 3 embryological layers: endoderm, mesoderm, and ectoderm [3]. The lesions in our case only show fat as germ layer component, which makes the diagnosis of a mature teratoma in an adult highly unlikely. Therefore, teratoma was not included in Table 1.

## Discussion

Intracranial lipomas are rare, benign tumors consisting of adipose tissue. Approximately 0.1%-1.3% of all intracranial tumors are lipomas, and <5% of intracranial lipomas are located at the Sylvian fissure [13]. Our patient presented with a coincidental finding of bilateral Sylvian fissure lipomas with angiomatous component. Interestingly, we found no previous reports of bilateral sylvian fissure lipomas. Nevertheless, bilateral intracranial lipomas have been documented in other locations (the cerebellopontine angle [[14], [15], [16], [17]], the choroid plexus [[18], [19], [20]] and the internal auditory canals [21]. Another striking feature of the lipomas in our patient is an angiomatous component, which is rare as well [22].

An MRI study of 42 patients showed the characteristic locations of intracranial lipomas to be: interhemispheric (45%), quadrigeminal/superior cerebellar (25%), suprasellar/interpeduncular (14%), cerebellopontine angle (9%) and Sylvian fissure (5%) [8]. Although the 2 lesions in our case are located at the Sylvian fissure, their position is superficial to the fissure, whereas all previously reported cases of Sylvian fissure lipomas are located deeply in the fissure.

The development of intracranial lipomas is believed to occur on embryonic level during formation of the subarachnoid cisterns. The neural crest and mesoderm surrounding the developing brain (meninx primitiva) may erroneously differentiate, maturing into lipoma. This process is not entirely understood [23].

Intracranial lipomas are usually asymptomatic, growing at the same rate as overall body growth. The lipomas can be discovered at any age and are usually incidental findings. Clinical symptoms associated with sylvian fissure lipomas include seizures, headache, and short-term memory problems [23]. Seizures are thought to be caused by compression of the mesiotemporal cortex due to the lipoma, or associated cortical dysplasia [22,24]. We identified 16 published cases of patients with Sylvian fissure lipomas with epileptic seizures, most of whom were treated successfully with antiepileptic medication. In some cases of intractable epilepsy, partial or complete removal of the lipoma has been performed [13,[24], [25], [26]], which generally led to favorable outcomes. However, surgical intervention may result in adverse events due to vascularization of the lipoma, adherence to the sylvian cortex, and proximity to the middle cerebral artery (MCA) and its branches [24,27,28]. Therefore, surgical treatment should be considered only when conservative treatment fails.

Headache has been described as a symptom of Sylvian fissure lipomas in 5 cases [22,23,[28], [29], [30]]. In 2 cases, partial [29] or complete [28] removal of the lipoma led to improvement, while the other 3 cases responded to conservative treatment [22,23,30]. Short-term memory problems are described in 1 case, although it remains unclear if they are directly related to the lipoma [23].

Some studies report associations between Sylvian fissure lipomas and middle cerebral artery (MCA) aneurysms or other vascular malformations [11,23,[31], [32], [33], [34]]. These vascular malformations include stretching of vessels in the regions of the mass, abnormal hypervascular networks branching out of the MCA, MCA dysplasia, and dilatation of the M2 and M3 segments of the MCA and angular artery [23]. Most cases with vascular malformations have been managed conservatively, although 1 case involved partial surgical removal of the lipoma and clipping of an associated aneurysm [29]. The authors of that case described the young age (26 years) of the patient as a reason to perform the surgery. However, no more considerations about the surgery were reported.

When our patient underwent her brain MRI, she was pregnant in her third trimester. We searched the literature for any reported association between intracranial lipomas and pregnancy but identified no such cases. Intracranial lipomas are congenital malformations that most often remain asymptomatic throughout life, and current evidence does not support a role for pregnancy in their pathogenesis or clinical course.

In conclusion, we present a unique case of bilateral Sylvian fissure lipomas with angiomatous component as an incidental finding. Generally, treatment of Sylvian fissure lipomas is unnecessary, due to their asymptomatic nature. Management involves patient education, reassuring the patient that the tumor is benign. Follow-up is usually unrequired but recommended in case of symptoms like headache and seizures. When conservative treatment is not effective, surgical treatment can be considered, weighing the individual risks and benefits. Cases like ours can aid in the prompt recognition of these benign lesions, thereby reducing resource expenditure and alleviating patient anxiety.

## Patient consent

The patient was informed about her diagnosis and its rarity, and she provided written informed consent to publish her data anonymously.
